# An Ethiopian Woman with an Incidental Finding During a Cesarean Section

**DOI:** 10.4269/ajtmh.14-0019

**Published:** 2014-12-03

**Authors:** Regev Cohen, Igor Igov

**Affiliations:** The Ruth and Bruce Rappaport Faculty of Medicine Technion, Infectious Diseases Unit, Sanz Medical Center, Laniado Hospital, Netanya, Israel; Surgery Department, Sanz Medical Center, Laniado Hospital, Netanya, Israel

## Abstract

This patient shows a rare phenomenon of schistosomal ova deposition on the serosal side of the small bowel without any pathology seen on the mucosal side of the small and large bowels. The patient was diagnosed accidentally during an elective cesarean section, when small nodules were seen on the small bowel surface.

A 26-year-old human immunodeficiency virus (HIV)-negative woman of Ethiopian descent was admitted for an elective cesarean section. During the operation, multiple nodules were noticed on the serosal surface of the small bowel (Figure 1

**Figure 1.**
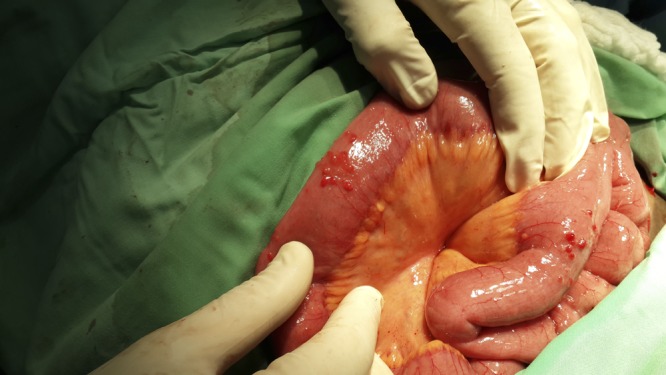
Multiple nodules on the surface of the small bowel.

). An excisional biopsy of a nodule showed granulomatous inflammation with eosinophils around an egg (Figure 2

**Figure 2.**
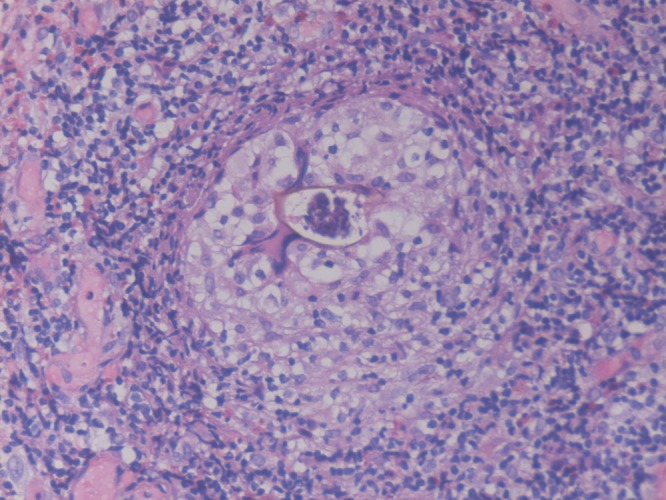
A granulomatous eosinophilic inflammation surrounding a Schistosomal ovum (H&E, 400×).

).

The patient immigrated to Israel 15 years before admission and was asymptomatic, except for mild chronic constipation and episodic dysuria. She denied hematuria, hematochezia, abdominal pain, tenesmus, and documented urinary tract infections. She could not recall an episode of rash or fever (representing Katayama fever). Physical examination and laboratory studies were normal, except for significant persistent eosinophilia of up to 2,200 cells/μL. Serology tests (immunoblot and FAST enzyme-linked immunosorbent assay [ELISA] in-house tests; Centers for Disease Control and Prevention) for *Schistosoma mansoni* were positive. Ultrasonography study of the abdomen was normal. Repeated stool examinations for ova were negative, and colonoscopy study was normal. Direct examination of rectal snip and terminal ileum biopsies were negative for ova, and pathology showed normal mucosa. Praziquantel was administered without side effects.

Peritoneal schistosomiasis is a rare, albeit under-reported phenomenon, which has been described with all three species of the parasite. It can be asymptomatic, like in the case presented, or cause ascites, weight loss, constipation, and infertility.^1^ Ova can reach the peritoneum by migration through the vessel wall or bloodstream embolization. Invasion can also occur through the fallopian tubes in cases of genital schistosomiasis.^2^ Ova located on the serosal side of the small bowel, especially with the absence of any pathology in the mucosal side of both the small bowel and the rectum, is unique.
